# Acute Effects of Percussive Massage Treatment on Drop Jump Performance and Achilles Tendon Stiffness

**DOI:** 10.3390/ijerph192215187

**Published:** 2022-11-17

**Authors:** Patryk Szymczyk, Kamil Węgrzynowicz, Robert Trybulski, Michał Spieszny, Paulina Ewertowska, Michał Wilk, Michał Krzysztofik

**Affiliations:** 1 Nutrition and Sports Performance Research Group, The Jerzy Kukuczka Academy of Physical Education in Katowice, 40-065 Katowice, Poland; 2 Provita Zory Medical Center, 44-240 Zory, Poland; 3 Department of Medical Sciences, The Wojciech Korfanty School of Economics, 40-659 Katowice, Poland; 4 Institute of Sports Sciences, University of Physical Education in Krakow, 31-571 Kraków, Poland; 5 Department of Physical Culture, Gdansk University of Physical Education and Sport, 80-336 Gdańsk, Poland; 6 Institute of Sport Sciences, The Jerzy Kukuczka Academy of Physical Education in Katowice, 40-065 Katowice, Poland; 7 Faculty of Physical Education and Sport, Charles University, 162 52 Prague, Czech Republic

**Keywords:** reactive strength index, power output, massage gun, fatigue, myotonometry

## Abstract

This study aimed to investigate the impact of Achilles tendon (AT) mechanical percussion massage (PM) on the passive stiffness of that tendon and subsequent drop jump kinematics. Eleven physically active participants performed two conditions in random order: (i) 60 s of PM applied to each AT (EXP) and (ii) no PM (CTRL). Measurements were performed 5 min before, immediately after, and 5 min following the completion of the PM. In the CTRL, measurements were performed at the same time point but no massage was applied. The two-way ANOVA indicated that there was no statistically significant interaction effect on contact time (*p* = 0.786), reactive strength index (*p* = 0.914), and relative peak power (*p* = 0.896). However, a statistically significant interaction on peak velocity (*p* = 0.046) and jump height (*p* = 0.03) was found. Despite that, there was no significant post-hoc comparisons for jump height, it slightly decreased 5 min post-PM (*p* = 0.136; ES = −0.25; Δ = −3.1%) compared with the CTRL condition (*p* = 1.00; ES = 0.11; Δ = +1.5%). Friedman’s test did not show significant differences in dominant (*p* = 0.073) and non-dominant limb (*p* = 0.091) AT stiffness. Although not significant, numerically, the dominant limb AT (*p* = 0.126; ES = −0.64; Δ = −7.8%) had a larger reduction in stiffness immediately post-PM compared with the non-dominant limb (*p* = 0.294; ES = −0.26; Δ = −3.6%). The results of this study indicated the temporary effect of PM on the reduction in tissue stiffness. Moreover, these findings show that a mechanical PM might slightly hinder subsequent explosive athletic performance.

## 1. Introduction

Percussion massage (PM) is a massage technique that combines elements of traditional massage and the vibration method [1]. Manual PM relies on applying a rapid, high-frequency, compressive striking with the tips of fingers or the edge of the hands to induce vibration throughout the selected part of the body [2,3]. However, it can also be applied by portable, easy-to-use, and inexpensive mechanical percussion devices which look similar to a gun. They allow us to set a chosen frequency, amplitude, and striking force and thus reduce the stress placed on the therapist during long-lasting manual PM or even allow us to perform it ourselves [1].

Despite high and growing mechanical PM popularity in amateur and professional sports, there is a paucity of studies investigating the underlying mechanism and post-treatment response and thus its acute and long-term impact on athletic performance [1]. The most frequently mentioned benefits of using PM include reducing pain and muscle spasms, increasing blood and lymphatic flow, increasing range of motion, and accelerating recovery [4,5,6]. Depending on the purpose, the PM is applied as a part of a warm-up, for instance, to increase the range of motion, or whether inter- and post-activity to accelerate recovery [4,7,8,9].

Studies carried out so far show that pre-exercise mechanical PM does not affect but may even have a positive effect on athletic performance. For example, Konrad et al. [9] indicated increased dorsiflexion range of motion without impact on a maximum voluntary contraction of plantar flexors after 5 min mechanical PM of calf muscles. Additionally, Garcia-Sillero et al. [7] found that using mechanical PM on the pectoralis major and minor during 3-min rest intervals increased the number of repetitions performed during four sets of bench press at 70% of one repetition maximum with a 30% velocity loss threshold. Interestingly, studies examining the use of pre-exercise whole-body vibration, which is somehow similar to PM, demonstrated acute, short-term athletic improvements via post-activation performance enhancement [10,11]. For instance, Cochrane et al. [10] showed a significantly higher rate of force development during patellar reflex activity after a 5 min static squat position on a vibrating platform compared to static squats without whole-body vibration or stationary cycling. Whereas Dallas et al. [11] found countermovement jump height augmentation 8 min after whole-body vibration (5 sets of 30 s squatting on the vibration platform) combined with 5 drop jumps, but not when those conditions were performed separately. The authors explained reported improvement due to the post-activation performance enhancement phenomenon, which is underpinned, among others, by an increase in blood flow and intramuscular temperature induced by the vibration intervention [12,13,14].

The mechanisms underlying the benefits of various massage techniques have not been extensively studied. For instance, an increase in the range of motion that has been noted after massage in some studies [9,15,16] might be partially explained by reduced passive stiffness of muscles and tendons. However, to the best of the authors’ knowledge, there is a paucity of data examining the impact of massage on the viscoelastic properties of muscles or tendons [15,16]. A study by Crommert et al. [15] found that massage reduced passive muscle stiffness, but this effect was short-lived and returned to baseline after 3 min. Nevertheless, the authors did not assess physical performance. In turn, Wang [16] reported an increase in gastrocnemius passive muscle stiffness immediately after the fatiguing protocol (calf raises until participants were unable to continue) and a significant decrease after the massage therapy day later. This was concomitant with a reduction in maximum voluntary contraction during plantar dorsiflexion directly post-exercising, which was returned a day after the massage treatment [16]. Therefore, reduction in the exercise-induced increase in passive stiffness may explain the accelerated recovery or, when used inter-activity, fatigue delaying effect due to the PM utilization.

To broaden the understanding of the acute effects of PM on tendon stiffness and athletic performance, this study aimed to investigate the impact of Achilles tendon mechanical PM as a part of a warm-up routine on drop jump selected variables and passive stiffness of that tendon. We hypothesized that mechanical PM would acutely reduce the Achilles tendon stiffness and improve drop jump performance, but it will return to baseline 5 min after intervention.

## 2. Materials and Methods

### 2.1. Experimental Approach to the Problem

**Figure 1:**
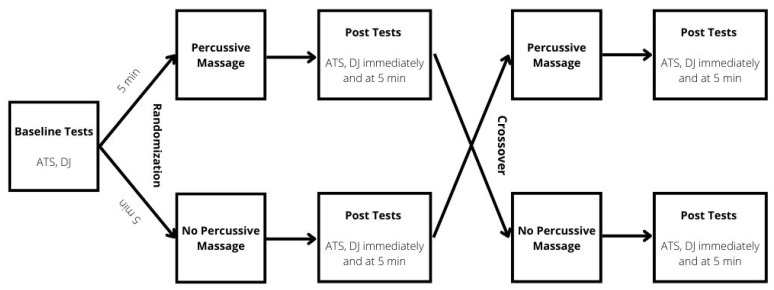
Study design flowchart. ATS–Achilles tendon stiffness; DJ–drop jump.

The experiment was performed following a randomized crossover design, where each participant performed two experimental sessions (a week apart) to assess the acute effects of PM treatment on drop jump selected kinematics variables and Achilles tendon stiffness. Participants were randomly assigned to two different conditions: (i) 60 s of PM applied to each Achilles tendon (EXP); (ii) no PM (CTRL). Measurements were performed 5 min before, immediately after, and 5 min following the completion of the PM. In the CTRL, measurements were performed at the same time point, but no massage was applied (Figure 1).

### 2.2. Subjects

Eleven physically active participants (8 males [24 ± 2 years, 78.3 ± 5.9 kg, 178 ± 7 cm] and 3 females: [all 24 years, 58.0 ± 8.2 kg, 163 ± 3 cm]) took part in this study. The inclusion criteria were as follows: (i) no history of Achilles tendon pain or injury that required surgery or caused them to seek medical treatment; (ii) regularly physically active for at least a year before the study; (iii) previously familiarized with the PM. Participants were instructed to maintain their usual dietary and sleep habits and not to use stimulants and alcoholic drinks throughout the study. Moreover, they were asked not to perform additional exercises 48 h before testing to avoid fatigue. Although some of the participants were females, the phase of the menstrual cycle during the experiment was not controlled. Participants were allowed to withdraw from the experiment at any time and were informed about the benefits and potential risks of the study before providing their written informed consent for participation. The Bioethics Committee for Scientific Research at the Academy of Physical Education in Katowice, Poland, approved the study protocol. The sample size was calculated a priori based on the following parameters “ANOVA, repeated measure, and within factors” as a statistical test (1 group of subjects, 2 experimental conditions, and 3 measurements); the statistical power of 0.8, an effect size of 0.66 and a significance level of 0.05, taking acute changes in passive stiffness after the massage as a reference variable [15]. A minimum sample size of 6 individuals was obtained (G*Power [version 3.1.9.2], Dusseldorf, Germany).

### 2.3. Familiarization Session

To avoid the influence of circadian rhythm on performance and stiffness, all study sessions were performed between 11:00 a.m. and 1:00 p.m. The session began by explaining the study procedure, but the expected study outcomes were not told. Moreover, leg dominance was determined by asking, “If you would shoot a ball on a target, which leg would you use to shoot the ball?”. Afterward, participants warmed-up with a 5-min run at 6 km/h on a treadmill. Then, they performed 10 repetitions of each exercise: bodyweight squats, split squats on each limb, side lunges on each limb, glute bridges, and calf raises. Then, they performed 2 sets of 2 repetitions of bilateral drop jumps from a 30 cm wooden box, followed by a 60 s Achilles tendon PM treatment of each limb.

### 2.4. Achilles Tendon Percussive Massage

After the warm-up (same as during the familiarization session) and baseline assessments (drop jump and Achilles tendon stiffness), the PM was applied. During the PM treatment, participants were lying prone on the massage table with the ankle in a neutral position (ankle joint dorsiflexion 0°). The PM was applied for 60 s to each Achilles tendon (in random order) approximately 6 cm above the tendon insertion (calcaneal tuberosity). During the control condition, no massage was applied, and the participants were prone for 2 min. The same investigator applied the PM using a mechanical device (Malatec, Poland). The device was set up to percussion frequency at 20 Hz, with a round tip of a 5.5 cm diameter. This PM duration and frequency were selected since health professionals match these values most frequently used [1]. Moreover, low frequencies of PM with a short duration were reported to improve muscular performance [17,18].

### 2.5. Measurement of Drop Jump Performance

The drop jump performance was measured using force plates (Force Decks, Vald Performance, Australia). This device has been previously confirmed as valid and reliable [19] for assessing vertical jump kinematics. Each participant performed two DJ without arm swings at pre-PM as a baseline, immediately after, and the 5th-minute post-PM. The participant started in the standing position with hands placed on the hips. To initiate the drop action, the participants were instructed to: “step off” the box one foot at a time and “jump up as fast as possible after contact with the ground, making sure that the jump is the highest possible”. The participant was instructed to perform the contact phase and landing phase on the force plate. The jump was invalid if the participant raised the feet during the jump flight, landed behind the force plate, or jumped off the box during the drop jump. The participant reset to the starting position after each jump, and the procedure was completed for a total of two jumps. The jump height, reactive strength index, peak velocity, and relative peak power were evaluated, and the best attempt in terms of jump height was kept for further analysis.

### 2.6. Measurement of Achilles Tendon Stiffness

The MyotonPRO, hand-held myometer (MyotonPRO, Myoton AS, Tallinn, Estonia) was used for the non-invasive assessment of Achilles tendon stiffness. The measurements were performed 6 cm above the tendon insertion (calcaneal tuberosity) at the neutral position (ankle joint dorsiflexion 0°) [20]. The Myoton’s accelerometer was set at 3200 Hz with an average value obtained from five consecutive measurements (0.4 N for 15 ms).

### 2.7. Statistical Analysis

All statistical analyses were performed using SPSS (version 25.0; SPSS, Inc., Chicago, IL, USA) and were shown as means with standard deviations (±SD) with their 95% confidence intervals (CI). Statistical significance was set at *p* < 0.05. The normality of data distribution was checked using Shapiro–Wilk tests. The relative (two-way mixed effects, absolute agreement, single rater intraclass correlation coefficient) and absolute (coefficient of variation) reliability were calculated. The thresholds for interpreting intraclass correlation coefficient results were: <0.5 “poor”, 0.5–0.75 “moderate”, <0.76–0.9 “good”, and >0.90 as “excellent” [21]. While the coefficient of variation results were: <10% “very good”, 10–20% “good”, <21–30% “acceptable”, >30% “not acceptable” [22]. The two-way ANOVAs (2 [EXP; CTRL] × 3 time-points [pre-PM; post-PM and 5 min post-PM]) were used to investigate the influence of PM on DJ variables. Since the normality for Achilles tendon stiffness data was not confirmed, related- samples of Friedman’s two-way ANOVA by ranks were used, and the effect size was estimated by Kendall’s coefficient of concordance. When a significant main effect or interaction was found, the post-hoc tests with Bonferroni correction were used to analyze the pairwise comparisons. The magnitude of mean differences was expressed with standardized ES (Hedges g). Thresholds for qualitative descriptors of Hedges g were interpreted as ≤0.20 “small”, 0.21–0.79 “medium”, and >0.80 as “large”.

## 3. Results

The Shapiro–Wilk tests did not indicate any violation of data distribution of drop jump variables; however, it was confirmed for Achilles tendon stiffness data. The ICC and CV results are presented in Table 1.

The two-way ANOVA indicated that there was no statistically significant interaction effect on contact time (F = 0.244; *p* = 0.786; η^2^ = 0.024), RSI (F = 0.09; *p* = 0.914; η^2^ = 0.009), relative peak power (F = 0.111; *p* = 0.896; η^2^ = 0.011). However, a statistically significant interaction on peak velocity (F = 3.596; *p* = 0.046; η^2^ = 0.294) and jump height (F = 4.196; *p* = 0.03; η^2^ = 0.296) was found. No main effects were statistically significant.

Although there were no significant post-hoc comparisons for jump height, it slightly decreased 5 min post-PM (*p* = 0.136; ES = −0.25; Δ = −3.1%) compared with the CTRL condition (*p* = 1.00; ES = 0.11; Δ = +1.5%) (Table 2).

**Figure 2:**
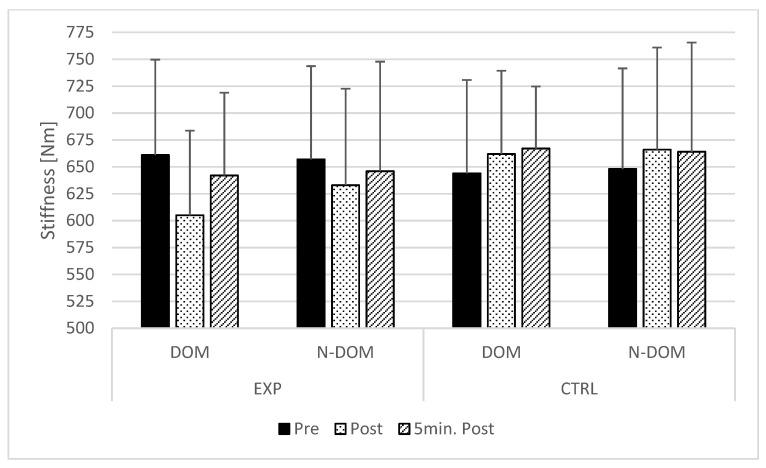
Comparisons of pre- and post-percussion massage Achilles tendon stiffness. DOM–dominant limb; N-DOM–nondominant limb; EXP–experimental condition; CTRL–control condition.

Friedman’s test did not show significant differences in the dominant (test = 10.078; *p* = 0.073; Kendall’s W = 0.183) and non-dominant limb (test = 9.479; *p* = 0.091; Kendall’s W = 0.172) Achilles tendon stiffness. Although not significant, numerically, the dominant limb Achilles tendon (*p* = 0.126; ES = −0.64; Δ = −7.8%) had a larger reduction in stiffness immediately post PM compared with the non-dominant limb (*p* = 0.294; ES = −0.26; Δ = −3.6%) (Figure 2).

## 4. Discussion

This study aimed to determine the effect of 60 s mechanical PM of the Achilles tendon on changes in its stiffness and on drop jump performed subsequently. The results showed that the drop jump performance measured immediately after the applied PM was not significantly impaired compared to pre-PM values; however, it should be noted that in 5 min after PM, the jump height decreased by ~3.1%. There was also a trend towards a decrease in Achilles tendon stiffness immediately after the PM, albeit not reaching a significant level, which returned almost to baseline value 5 min after the PM. The results of this study support the earlier report of the temporary effect of massage on the reduction in tissue stiffness [15]. Moreover, these findings indicate that a mechanical PM as a part of a warm-up might hinder explosive athletic performance.

The assumed hypothesis was partially confirmed as there was a trend to reduce Achilles tendon passive stiffness immediately after the mechanical PM with no effect on jumping performance; however, it did not reach a statistically significant level. Nevertheless, 5 min after PM, the Achilles tendon passive stiffness returned to the baseline value with a concomitant and insignificant decrease in drop jump height. Regarding the influence of mechanical PM on physical fitness, these results are partially consistent with the reports to date [7,9]. A study by Konrad et al. [9] reveals no effect of 5 min mechanical PM on calf muscles on plantar flexor strength. While Garcia-Sillero et al. [7] even showed increased bench press repetitions when the mechanical PM was applied between sets. Moreover, McKechnie et al. [23] showed that the manual tapotement massage, which is a similar technique to PM, of 3 min (of each calf muscle) did not affect the drop jump performed immediately after. Nevertheless, it should be noted that none of these studies assessed performance later after the completion of the PM. The present study also did not show the effect of PM on the drop jump performed right after, but 5 min post-PM, a slight decrease in the drop jump height was noted. Even though in this study, a shorter duration of mechanical PM was used compared to the previously mentioned studies.

In addition, despite those previous studies indicating that whole-body vibration, which is a similar technique to PM, effectively increases muscular performance by eliciting post-activation performance enhancement [10,11], the mechanical PM applied in this study was ineffective in inducing this phenomenon. Nevertheless, the reason may be the slight effect of the used PM on the muscles. The post-activation performance enhancement effect is associated with an improvement in the muscle contraction capabilities due to prior muscle activity [24], while the PM in this study was applied to the tendon. Moreover, the absence of a post-activation performance enhancement may also be related to changes in temperature and/or blood flow which underpin this phenomenon [13]. It was not actually evaluated, but it is possible that 60 s mechanical PM is insufficient to induce a significant increase in temperature and/or blood flow that would also be maintained 5 min after PM.

The results of this study partially confirm previous reports from Crommert et al. [15] on immediate reduction and quick return of passive stiffness to the baseline value after the applied massage. Although there was no significant decrease in Achilles tendon passive stiffness in this study, the downward trend was visible. The lack of such a considerable effect on passive stiffness might be explained by the fact that in this study, the massage lasted 60 s, while in the study by Crommert et al. [15] it was 7 min. However, Crommert et al. [15] used a combination of other massage techniques, including effleurage, petrissage, and deep, circular friction. Furthermore, according to the best of the authors’ knowledge, there are no studies available to assess the effect of mechanical PM on passive tissue stiffness. Nevertheless, it has been speculated that the increased range of motion after mechanical PM may have been partially obtained due to a reduction in passive stiffness [9]. Unfortunately, the authors did not evaluate how long this effect is maintained.

Findings from the current study may indicate that 60 s of mechanical PM is insufficient to reduce the Achilles tendon’s stiffness significantly. However, it should be noted that there are no precise guidelines for the optimal duration of mechanical PM, and recent reports indicate that healthcare professionals prefer a total treatment time between 30 s to 3 min [1]. This points to the need for future empirical studies to assess the effect of different durations of mechanical PM and to establish how long post-PM effects are maintained in terms of stiffness and physical performance.

Interestingly and worth mentioning is that the trend to reduce the passive stiffness of the Achilles tendon was more noticeable in the tendon of the dominant leg. However, the results of this study did not show significant differences in the baseline level of passive stiffness between the limbs. This may indicate the influence of lateralization and the efficiency of mechanical PM in reducing passive stiffness. Perhaps the reason lies in the differences in blood flow. For example, Kagaya et al. [25] have shown that post-exercise blood flow is higher due to the enlarged vessel diameter during diastole compared to the non-dominant limb. However, it should be noted that this study included female tennis players who suspected the symmetry of the limbs was more impaired than the participants in this study. Kagaya et al. [25] showed significantly greater muscle thickness and muscle strength in the forearm of the dominant limb than in the non-dominant limb. Whereas in this study, no assessment of symmetry was made. Nevertheless, these results provide the basis for future research to investigate the potential effects of lateralization on PM efficiency, along with an evaluation of possible mechanisms such as the assessment of stiffness, blood flow, and temperature variation.

The most important limitation of this study is the small sample size; therefore, further, more extensive studies on this issue are needed to clarify the effects of mechanical PM on passive tissue stiffness and athletic performance. In addition, only a single mechanical PM setting was used on the tendon, and only an explosive athletic task was tested; therefore, extrapolating these results for other conditions should be performed with caution.

## 5. Conclusions

Coaches and practitioners should be aware that although the short-lasting mechanical PM of the Achilles tendon may slightly decrease the passive stiffness of this tendon, that effect is short-term. Moreover, it may contribute to a slight reduction in the height of subsequent drop jumps. Therefore, we would like to point out that the application of mechanical PM as a part of the warm-up has to be considered, especially before performing explosive athletic activities.

## Figures and Tables

**Table 1:** Intersession reliability of the drop jump performance, Achilles tendon stiffness.

Variables	ICC [95% CI]	CV [%]
Jump Height	0.91 (0.68 to 0.98)	11%
Contact Time	0.92 (0.70 to 0.98)	4%
Reactive Strength Index	0.85 (0.62 to 0.9)	10%
Relative Peak Power	0.84 (0.66 to 0.91)	7%
Peak Velocity	0.92 (0.69 to 0.98)	2%
Dominant Achilles Tendon Stiffness	0.96 (0.88 to 0.99)	3%
Non-dominant Achilles Tendon Stiffness	0.92 (0.71 to 0.98)	4%

ICC–intraclass correlation coefficient; CV–coefficient of variation.

**Table 2:** Comparisons of pre- and post-PM drop jump and Achilles tendon stiffness.

Variable	EXP			CTRL		
	Pre	Post	5 min. Post	Pre	Post	5 min. Post
Jump Height [cm]	36.9 ± 5.5 (33.2 to 40.6)	37.3 ± 5.1 (33.8 to 40.7)	35.6 ± 4.6 (32.5 to 38.7)	36.1 ± 4.4 (33.1 to 39.0)	36.2 ± 4.8 (33.0 to 39.4)	36.6 ± 4.3 (33.6 to 39.5)
Contact Time [ms]	0.383 ± 0.128 (0.297 to 0.469)	0.385 ± 0.118 (0.306 to 0.464)	0.374 ± 0.118 (0.295 to 0.454)	0.366 ± 0.132 (0.278 to 0.455)	0.380 ± 0.127 (0.295 to 0.465)	0.373 ± 0.113 (0.297 to 0.449)
Reactive Strength Index	1.05 ± 0.29 (0.86 to 1.24)	1.04 ± 0.27 (0.85 to 1.22)	1.03 ± 0.28 (0.84 to 1.22)	1.09 ± 0.31 (0.88 to 1.29)	1.04 ± 0.33 (0.82 to 1.27)	1.05 ± 0.28 (0.86 to 1.25)
Relative Peak Power [W/kg]	113.6 ± 16.0 (102.9 to 124.3)	111.2 ± 13.9 (101.8 to 120.5)	109.2 ± 14.5 (99.4 to 118.9)	114.7 ± 18.1 (102.6 to 126.9)	111.1 ± 19.4 (98.1 to 124.2)	111.7 ± 15.6 (101.2 to 122.2)
Peak Velocity [m/s]	2.81 ± 0.20 (2.68 to 2.95)	2.82 ± 0.19 (2.70 to 2.95)	2.76 ± 0.17 (2.65 to 2.88)	2.78 ± 0.17 (2.67 to 2.90)	2.79 ± 0.18 (2.66 to 2.91)	2.80 ± 0.17 (2.68 to 2.91)

PM–percussion massage; EXP–experimental condition; CTRL–control condition.

## Data Availability

The datasets used and analyzed during the current study are available from the corresponding author upon reasonable request.
